# Chondro-Osseous Respiratory Epithelial Adenomatoid Hamartoma (COREAH): Case Report and Literature Review

**DOI:** 10.1155/2019/5247091

**Published:** 2019-07-25

**Authors:** Andrew Daniel, Eugene Wong, Joyce Ho, Narinder Singh

**Affiliations:** Westmead Hospital, Westmead, NSW, Australia

## Abstract

Chondro-osseous respiratory epithelial adenomatoid hamartoma (COREAH) is an extremely rare lesion of the nasal cavity with only 11 reported cases in the literature. COREAH is of interest as it may be easily mistaken for other diseases of the nasal cavity with higher morbidity, which require significantly different management strategies. We report, to the best of our knowledge, the oldest documented case of COREAH in the literature: an 83-year-old female who presented with headaches and was found to have a posterior nasal septal lesion. Uniquely, the patient had sequential scans performed 1 year apart demonstrating only minor interval growth. We describe our experience in managing a patient with COREAH and review the world literature, to better define aspects of the history, presentation, and investigations that may allow differentiation from more sinister disease.

## 1. Introduction

Chondro-osseous respiratory epithelial adenomatoid hamartoma (COREAH) of the sinonasal tract is an exceedingly rare benign entity with only 11 cases reported in the literature. It often presents with similar clinical history and macroscopic and radiological appearance to that of malignant disease. We present a case report of an 83-year-old female who presented to our facility with COREAH and discuss its key characteristics. To the best of our knowledge, this represents the oldest documented case of COREAH in the literature.

## 2. Clinical Case

An 83-year-old Filipino female was referred to our clinic with a 3-year history of headaches. She also reported intermittent perioral paraesthesia but denied epistaxis, rhinorrhoea, anosmia, or facial pain. She had a past medical history of hypertension managed on perindopril and previous transient ischaemic attack (TIA). She was a lifelong nonsmoker and had no significant history of alcohol use.

On flexible nasendoscopic examination, a left posterior septal mucosal-covered, polypoid, pedunculated mass without ulceration was identified and found to extend into the nasopharynx. The right nasal passage was clear with no extension through the septum.

**Figure 1:**
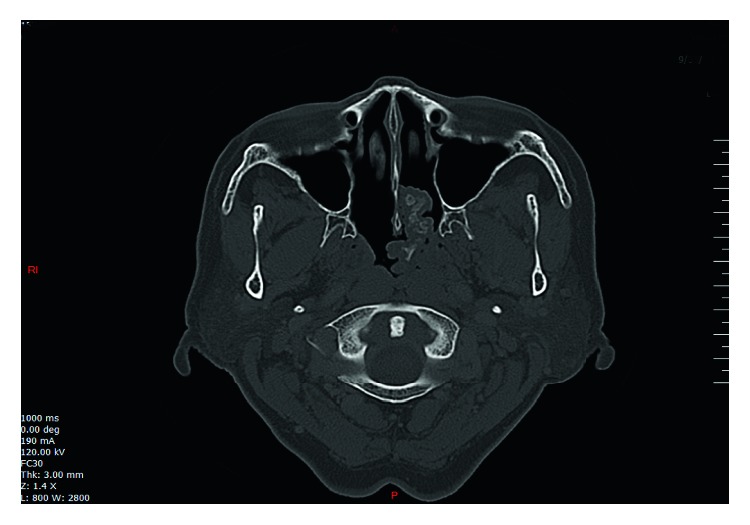
CT brain demonstrating left nasal mass with nasopharyngeal extension.

A high-resolution computed tomography (CT) of the paranasal sinuses demonstrated a left nasal mass measuring approximately 3.4 × 4.1 cm (Figure 1).

Examination under anaesthesia (EUA) and biopsy were recommended. However, the patient was unwilling to proceed and declined further investigations or treatment. She was subsequently lost to follow-up. One year later, the patient re-presented to the emergency department with an acute onset of coughing and mild epistaxis. Clinical examination demonstrated that the lesion had marginally grown since previous review, now crossing the midline posterior to the septum.

**Figure 2:**
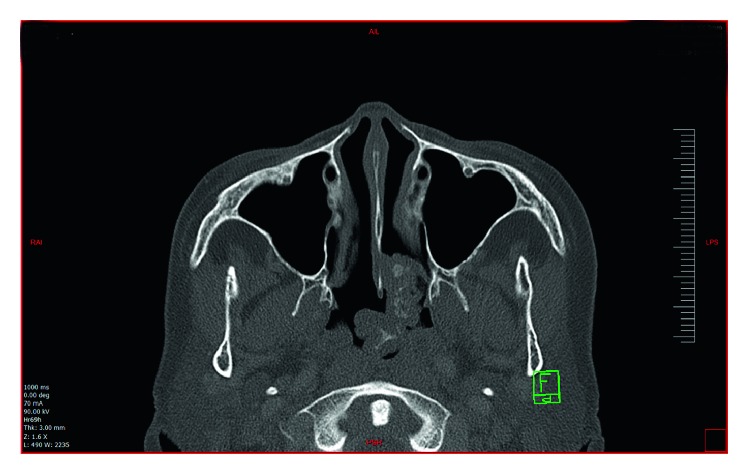
Posterior nasal mass with calcified core. CT performed 1 year following the previous scan (Figure 1).

**Figure 3:**
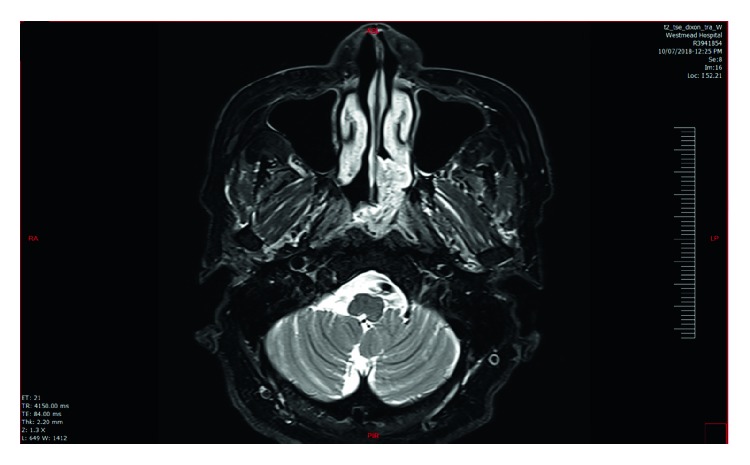
T2 MRI brain showing the mass with cerebriform appearance with extension into right posterior nasal space.

Repeat CT of the paranasal sinuses demonstrated an irregular 4.8 × 5.2 cm polypoid mass originating from the posterior nasal septum and projecting into the left side of the nasopharynx. The mass had a central core of high-density material, with an outer soft-tissue layer, and did not enhance with contrast (Figure 2). Magnetic resonance imaging (MRI) demonstrated a slightly heterogenous though predominantly T2 hyperintense and T1 hypointense mass. Gadolinium contrast demonstrated a heterogenous, cerebriform appearance (Figure 3).

In view of the slow growth of the lesion and central high-density, a presumptive diagnosis of a rhinolith was made, while neoplasia was considered less likely.

## 3. Management

Intraoperative findings demonstrated a left sided, mucosal-covered, polypoid septal lesion extending from the upper half of the choana with a narrow pedicle to the left clival mucosa and inferior edge of the superior turbinate. The lesion was removed completely through an endoscopic approach. The patient remains disease-free after 6 months.

## 4. Pathology

**Figure 4:**
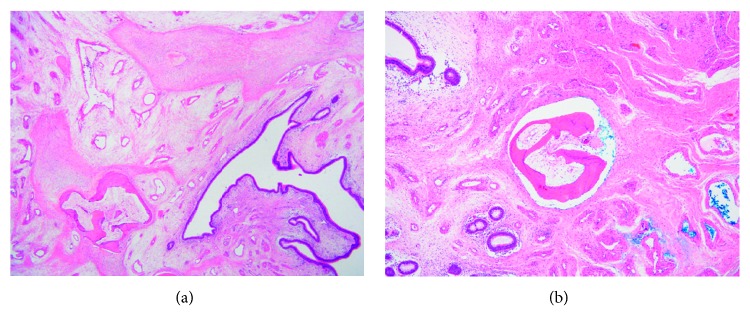
(a, b) Photomicrographs demonstrating invaginated ductal structures/glands within the submucosa, vital bone (some of which has marrow space with fat), and nodular collections of fibroblasts and collagen. Normal respiratory epithelium is seen with proliferation.

Histopathology demonstrated respiratory-type epithelium and focal lymphocytic epitheliotropism noted, without dysplasia. The specimens were observed to have a central area of supporting bone, and the underlying lamina propria and submucosal connective tissue were oedematous with patchy collections of chronic inflammation (Figures 4(a) and 4(b)). The above findings supported a diagnosis of a hamartoma consistent with COREAH.

## 5. Discussion

A hamartoma is defined as a nonneoplastic mass of disorganised tissue, indigenous to the particular site where it is found, and traditionally considered to be a developmental malformation [1].

Respiratory epithelial adenomatoid hamartoma (REAH) has long been identified as a solely epithelial hamartoma of the sinonasal tract, while nasal chondromesenchymal hamartoma (NCMH) has been identified as a solely mesenchymal hamartoma. It has been suggested that COREAH lies in the spectrum between REAH and the DICER 1 familial disorder NCMH as it encompasses elements of each entity, both epithelial and mesenchymal [2]. However, it should be noted that to date, COREAH has not been observed in DICER 1 patients and thus may represent a completely different entity.

NCMH was first described in 1998 by McDermott et al. in a case series of 7 patients in which the authors identified a predominately infant population susceptible to the disease [3]. NCMH is considered to be a familial disorder associated with DICER 1 germline mutation similar to pleuropulmonary blastoma and other ovarian sex cord-stromal tumours [4].

REAH was first described in 1995 by Wenig and Heffner in a series of 31 cases of sinonasal tract lesions, including one case that contained osseous metaplasia [5]. Subsequently, in 1996, Adair et al. coined the term COREAH for this particular lesion [6]. REAH typically occurs in adults with strong male predominance in the fifth and sixth decades of life and is associated with inflammatory nasal polyps. It has a similar histological appearance to COREAH, with proliferation of glandular tissue. However, it lacks the mesenchymal characteristics seen in COREAH [7].

A formal review of the literature highlights the rarity of COREAH, with only 11 previous cases reported [5, 8–16].

Our case is the most elderly reported case to date of COREAH and presented with symptoms of headache and epistaxis rather than obstruction, fullness, or anosmia. Furthermore, the patient had 2 consecutive presentations, 1 year apart, demonstrating growth of the lesion clinically and radiologically.

The aetiology of COREAH is unclear and may be secondary to embryological malformation, inflammation, or neoplasia. Embryological malformation is believed to be the origin of most hamartomas in other areas of the body [14]. Two previous cases of COREAH in a 3-year-old boy and a 7-year-old female support this possible congenital aetiology [2, 8]. Unlike NCMH however, COREAH is not associated with other genetic disease processes and often presents at a later stage in life, making a congenital aetiology less likely. Our patient presented at age 83 with only 3 years of symptoms and interval changes on imaging, which supports a noncongenital cause for COREAH.

REAH has been reported to be related to chronic sinusitis and nasal polyps, which has led some authors to suggest an inflammatory aetiology for COREAH given the close histological characteristics. Roffman et al. present a case of a 59-year-old man with unilateral COREAH, nasal polyps, and REAH in the opposite nasal cavity suggestive of an inflammatory aetiology for COREAH [11]. A study by Gu et al. found that the expression of T-helper-type 9 cells and interleukin-9, a proinflammatory protein, was significantly greater in patients with REAH compared to controls. Their study further added to the theory that the inflammatory response may play a central role in the development of REAH. By extrapolation, this may also suggest that COREAH is associated with a prolonged proinflammatory environment [17].

However, a review of the other 10 cases of COREAH shows no evidence of patients reporting chronic rhinosinusitis (CRS) symptoms or nasal polyps, similar to the current case, with nasal obstruction being the most common presentation.

Despite this, most of the reported cases demonstrate changes typically seen in inflammation within the lesion itself, including stromal oedema, vascular and fibroblastic proliferation, and polypoid growth [13]. A 64-year-old female reported by Chatzopoulos et al. [15] showed microscopy consistent with intense oedema, moderate chronic inflammatory infiltrates, and bizarre stromal cells which have also been seen in inflammatory nasal polyps. Our case presented histologically with lymphocytic epitheliotropism and patchy areas of chronic inflammation.

Neoplastic changes have also been attributed to the formation of REAH in which one study found molecular changes in two tumour suppressor genes associated with the hamartoma [18]. To date, no molecular studies have investigated the formation of COREAH; however, it is noted that COREAH does not have malignant potential and is self-limiting.

As most cases of COREAH present with nasal obstruction and clinical features of a unilateral mass, the possibility of neoplasia is often raised. In REAH, histopathology can demonstrate features similar to sinonasal adenocarcinoma and inverted papilloma, which could result in misdiagnosis and unnecessarily destructive surgical management. The chondro-osseous component in COREAH makes misdiagnosis less likely than with REAH, but vigilance must still be maintained.

Fang et al. [2] compared 6 previous case studies of COREAH, [8, 10–13], including parameters of age, sex, origin, presentation, duration of symptoms, and follow-up period. The study found clinical similarities in presentation of nasal obstruction, feeling of fullness, and anosmia. Patients of all ages were reported (3–59) with no significant gender preponderance. The authors found that although CT and MRI were utilised to identify the presence of the lesion, no similar characteristic signals were identified given the different elements of the hamartomas.

The MRI of the patient described in the case study showed radiological “cerebriform appearance” which was in keeping with classical MRI findings of inverted papilloma [19]. Interestingly, we present the first reported case of COREAH presenting with these similar characteristics on MRI imaging and suggest that it should be included into the differentials in MRI reporting when this characteristic feature is present in sinonasal masses.

In conclusion, COREAH is an important lesion to identify for clinicians and pathologists given its benign nature as well as similarities with more sinister pathology in the nasal cavity. It is important to clearly identify these lesions to avoid unnecessary surgical intervention.

## Additional Points

(i) We present the most elderly documented case of COREAH and explore its implications on the cause and management of the disease process. (ii) COREAH is a benign disease highlighted in its slow growth, lack of malignant potential, and recurrence. (iii) COREAH presents similarly to other malignant diseases including sinonasal adenocarcinoma and inverted papilloma, and so knowledge of COREAH can help avoid unnecessary surgical intervention on patients.
